# Aspects cliniques des cancers bronchopulmonaires primitifs au service d'oncologie du CHUA-HUJRA Antananarivo

**DOI:** 10.11604/pamj.2015.22.271.7931

**Published:** 2015-11-20

**Authors:** Valéry Refeno, Nomeharisoa Rodrigue Emile Hasiniatsy, Ny Ony Tiana Florence Andrianandrasana, Andriatsihoarana Voahary Nasandratriniavo Ramahandrisoa, Jean Marc Rakotonarivo, Joée Larissa Maevazaka, Hanitrala Jean Louis Rakotovao, Florine Rafaramino

**Affiliations:** 1 Faculté de Médecine Antananarivo, Antananarivo-101, Madagascar

**Keywords:** Cancer bronchopulmonaire, cliniques, Madagascar, primitif, lung cancer, clinics, Madagascar, primitive

## Abstract

Le retard de diagnostic des cancers broncho-pulmonaires est l'une des sources du retard de leur prise en charge dans les pays en développement. A notre connaissance, l'aspect clinique des cancers broncho-pulmonaires au Centre Hospitalier Universitaire d'Antananarivo-Hôpital Universitaire Joseph Ravoahangy Andrianavalona (CHUA-HUJRA) n'a jamais été étudié. L'objectif était de décrire les aspects cliniques des cancers broncho-pulmonaires primitifs dans le plus grand centre de cancérologie de Madagascar. C'est une étude rétrospective et descriptive des patients atteints de cancers broncho-pulmonaires primitifs vus au service d'oncologie du CHUA-HUJRA du 1er janvier 2008 au 31 décembre 2013. Nous avons recensé 101 patients (80 hommes et 21 femmes). Les circonstances de découverte sont principalement la toux chronique (n = 29), la dyspnée (n = 16) et l'association d'une hémoptysie à la toux chronique (n = 12). Soixante et onze patients avaient un index de performans status ≥ à 2 au moment du diagnostic. On a retrouvé des bacilles de Koch actives dans le crachat de deux patients. Le délai moyen entre l'apparition des premiers signes et la première consultation était de 11 mois. Le délai moyen entre la première consultation et le diagnostic anatomopathologique était de 3 mois. Le cancer broncho-pulmonaire peut avoir des manifestations cliniques non spécifiques parfois trompeuses qui peuvent retarder leur prise en charge. De ce fait, il doit être recherché devant tout signe respiratoire persistant. Par ailleurs, le délai de prise en charge pré-hospitalière et hospitalière de ces cancers doit être amélioré.

## Introduction

Le cancer broncho-pulmonaire primitif est actuellement la première cause de mortalité par cancer dans le monde. Elle représente 18,2% du total des décès par cancer dans le monde en 2008 [1]. C'est un fléau mondial né de la mondialisation du tabagisme [2]. Autrefois considéré comme apanage des pays industrialisés, il est aujourd'hui en recrudescence dans les pays en développement dont les pays du continent africain. La prévalence des cancers broncho-pulmonaires est sous-estimée en Afrique du fait de l'absence des moyens diagnostiques, de l'absence de recueil de données fiable et parce qu'elle est confondue avec la tuberculose avec laquelle elle partage des similitudes cliniques [3, 4]. A Madagascar, les cancers broncho-pulmonaires représentaient 2,97% des nouveaux cas de cancers rencontrés dans le service de l'oncologie du Centre Hospitalier Universitaire ‘ Hôpital Universitaire Joseph Ravoahangy Andrianavalona (CHUA-HUJRA) de 2009 à 2010 alors qu'il était le seul centre de cancérologie à Madagascar avant l'année 2011 [5]. A notre connaissance, aucune étude portant sur l'aspect clinique des cancers bronchopulmonaire n'a encore été réalisée au service d'oncologie du CHUA-HUJRA. Pourtant, les symptomatologies variables et non spécifiques de ces cancers peuvent être source de retard diagnostique, de confusion ou de négligence [6]. Ainsi, l'objectif de notre travail est d’étudier l'aspect clinique des cancers bronchopulmonaires dans le plus grand centre de cancérologie de Madagascar.

## Méthodes

Il s'agit d'une étude rétrospective et descriptive des patients atteints de cancers broncho-pulmonaires primitifs vus au service d'oncologie du Centre Hospitalier Universitaire-Hôpital Universitaire Joseph Ravoahangy Andrianavalona (CHUA-HUJRA) durant la période allant du 1^er^ janvier 2008 au 31 décembre 2013 soit sur une période de 6 ans. Nous avons inclus les patients ayant une preuve anatomo-pathologique et/ou cytologique de cancer broncho-pulmonaire primitif et dont les dossiers sont complets. Nous avons étudiés pour chaque patient les paramètres suivants: l'index de performans status, les circonstances de découverte, le délai entre la date des premiers symptômes et la date de la première consultation médicale, le délai entre la première consultation médicale et la date du diagnostic anatomopathologique et enfin la date entre les premiers symptômes et la date du diagnostic anatomopathologique.

## Résultats

**Figure 1:**
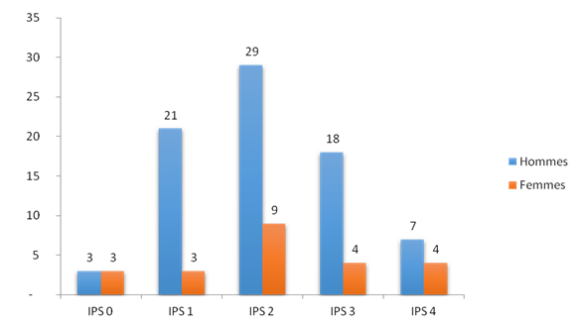
Répartition selon l'index de performans status et selon le genre

**Figure 2:**
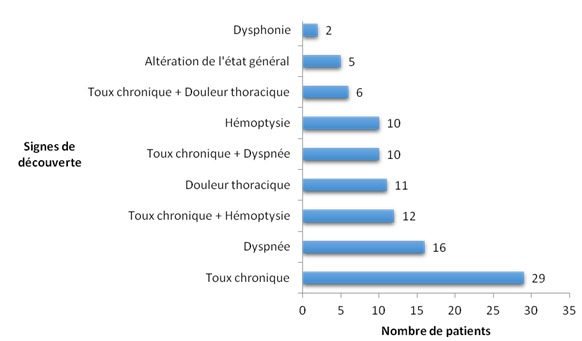
Répartition des patients selon les signes de découverte

Nous avons colligé 101 nouveaux cas (80 hommes et 21 femmes) de cancers bronchiques pendant la période de janvier 2008 à décembre 2013. L’âge moyen des patients était de 56,18 ± 12,49 ans. Les 82% des patients appartenaient à l'ethnie Merina. A l'entrée, 71 patients ont eu un score de performans status supérieur ou égal à 2. L'on a également noté une prédominance masculine de l'altération de l’état générale (Figure 1). La toux chronique (n= 29) constituait la première circonstance de découverte des cancers broncho-pulmonaires primitifs suivie par la dyspnée (n = 16) et l'association toux chronique et hémoptysie (n= 12) tels que représentés dans la Figure 2. Deux de nos patients présentaient des bacilles de Koch dans leurs crachats. Le délai moyen entre les premiers symptômes et la première consultation a été de 11 mois. Quarante sept patients ont consulté dans les 3 mois après les premiers signes. Le délai moyen entre la première consultation et le diagnostic anatomopathologique a été de 3 mois. Le diagnostic a été posé au bout de 1 à 4 mois après les premiers signes pour 52 patients. Enfin, le délai moyen entre les premiers symptômes et le diagnostic anatomopathologique a été de 14 mois.

## Discussion

Tous nos patients ont été symptomatiques au moment du diagnostic. Les signes d'appels les plus fréquemment rencontrés étaient la toux chronique seule (n = 29), la dyspnée seule (n = 16), l'association toux chronique et hémoptysie (n = 12) et la douleur thoracique seule (n = 11). Comme dans notre cas, la toux chronique constitue la symptomatologie la plus fréquemment retrouvée dans la littérature [6]. Des études réalisées en Afrique notamment au Mali et au Maroc ont retrouvés une fréquence plus importante des douleurs thoraciques, respectivement de 72,2% et de 71% [4]. Ce signe est reflet d'une extension locorégionale de la maladie, donc d'un diagnostic tardif [6] comme tel est le cas dans de nombreux pays en voie de développement. Aucun de nos patients n'a été diagnostiqué au stade asymptomatique, ce qui diverge avec la plupart des données de la littérature. Selon Bizieux-Thaminy et al, 1 à 6% des cancers bronchiques sont découverts fortuitement par la réalisation d'une radiographie thoracique systématique [7]. De même, selon l’étude de De Pierrot et al, 32% des femmes et 20% des hommes de son échantillon n'ont pas été symptomatiques au moment du diagnostic [8]. L'absence de découverte fortuite pourrait s'expliquer par l'insuffisance des moyens d'exploration paracliniques dans les zones rurales mais aussi par la réticence des médecins à prescrire des examens complémentaires devant les signes peu évocateurs. Une meilleure accessibilité aux examens anatomopathologiques et d'imagerie ainsi qu'une formation médicale continue des médecins pourrait accélérer le diagnostic précoce des cancers bronchiques. De nombreuses études ont retrouvé la présence d'hippocratisme digital chez les personnes atteintes de cancer bronchopulmonaire [9, 10]. Nous n'avons pas tenu compte de cette variable lors de notre étude. Du fait que Madagascar soit un pays à faibles ressources où les explorations paracliniques ne sont pas à la portée de tous les patients, ce signe d'inspection, simple, ne devrait pas être négligé et doit-être recherché systématiquement. La tuberculose, encore endémique dans les pays en voie de développement est source d'errance diagnostique et induit le clinicien en erreur devant le diagnostic du cancer bronchique. En effet, les signes d'imprégnation tuberculeuse sont essentiellement des signes respiratoires tels que la toux chronique et l'hémoptysie ainsi que l'altération de l’état général [4, 11]. Ces signes sont superposables à ceux retrouvés dans notre étude. La difficulté diagnostique se pose car la tuberculose peut simuler le cancer tant sur la symptomatologie que sur l'imagerie [12]. La radiographie du thorax seule ne permet pas de distinguer les lésions tuberculeuses des lésions cancéreuses. La réalisation d'une tomodensitométrie thoracique et/ou d'un examen histologique ou cytologique s'avère importante afin de pouvoir différencier ses deux pathologies [13].

Cependant, il faut souligner que l'association entre une tuberculose pulmonaire et un cancer bronchique a été rapportée par plusieurs auteurs. En effet, 1 à 2% des cancers bronchiques seraient associés à la tuberculose tandis que 1 à 5% des tuberculoses seraient associés à un cancer bronchique [14]. Même si le plus souvent la découverte des deux pathologies se fait de façon séquentielle, des rares observations rapportant la découverte concomitante d'un cancer bronchique et d'une tuberculose ont été publiées [13, 15]. Ceci appuie le fait que nous ayons retrouvé deux patients présentant à la fois un cancer bronchique et des bacilles tuberculeux actifs retrouvés dans leur crachat. Ces patients nécessiteront une prise en charge personnalisée décidée en réunion de concertation pluridisciplinaire afin de coordonner la prise en charge de ses deux entités [13]. Les praticiens Malgaches devront prendre en compte la possibilité d'association de ces deux pathologies fréquentes et graves. Dans notre étude, plus de la moitié des patients étaient en mauvais état général avec un score de performans status supérieur ou égal à 2 (n = 71 soit 70,29%). L’étude de Kaptue au Mali en Afrique a également retrouvé une altération générale chez 75% de ses patients atteints de cancers broncho-pulmonaires [4]. D'autres études viennent appuyer cette fréquence de l'altération de l’état général au cours des cancers bronchique retrouvés en Afrique [3]. La tendance inverse s'observe dans les pays nanti. En effet, selon l'Etude KBP-2010-CPHG, 68,8% des patients atteints de cancer bronchopulmonaires avaient un score de performans status entre 0 et 1 [16]. Nous avons constaté une prédominance masculine de l'altération de l’état générale. Cette prédominance pourrait s'expliquer par la prédominance du genre masculin dans notre échantillon (80 hommes contre 21 femmes). L’étude de Cadelis menée en Guadeloupe n'a pas retrouvé de prédominance du genre [17]. Cette prévalence de l'altération de l’état général dans notre étude pourrait s'expliquer d'une part par le fait que les patients ne viennent consulter que lorsque leur état général s'altère et d'une autre du fait du retard du diagnostic de la maladie. Les délais d'accès aux soins permettent de juger de l'efficacité du système sanitaire dans son ensemble sur un territoire. Ils permettent aussi de mettre en évidence des inégalités qui peuvent nécessiter une correction de la part des pouvoirs publics. Par ailleurs, les délais peuvent aussi avoir un impact sur la progression de la maladie [18, 19]. Dans notre travail, le délai moyen entre les premiers symptômes et la première consultation médicale a été de 11 mois, tandis que l'espace de temps moyen entre la première consultation et le diagnostic a été de 3 mois. Enfin, le délai moyen entre les premiers symptômes et le diagnostic a été de 14 mois. Ce dernier délai est moins long dans les études effectuées dans d'autres centres. Le délai moyen entre les premiers symptômes et le diagnostic a été de 2,4 mois dans l’étude KBP-2000 [20] et de 1 mois +/- 9 jours selon l’étude de Cadelis en Guadeloupe [17]. Ce délai allongé dans notre étude pourrait avoir plusieurs explications: d'abord les distances à parcourir vers les centres de santé sont longues et le réseau routier impraticable dans certaines régions. En outre la caractère banal des symptômes entraine le retard diagnostique [6]. La dernière hypothèse est l'accessibilité aux centres d'imagerie et d'anatomopathologie dont les services sont payants. Les études menées dans les centres d'oncologie à Madagascar se heurtent souvent au problème financier. Une fois le diagnostic posé, le patient prend souvent beaucoup de temps à trouver les moyens financiers en vue du traitement. Parfois même le traitement est arrêté par faute de moyens. Ce manque de moyens entraine des difficultés pour recueillir la date du 1er jour de traitement et les modalités thérapeutiques ainsi que le résultat thérapeutique. L'attribution des budgets spéciaux aux pathologies cancéreuses par l'Etat pourrait améliorer la prise en charge des patients.

## Conclusion

Le cancer broncho-pulmonaire peut avoir des manifestations non spécifiques parfois trompeuses qui retardent sa prise en charge spécifique. De ce fait, il doit-être recherché systématiquement devant tous signes respiratoires persistants en particuliers ceux évocateurs d'une tuberculose pulmonaire avec laquelle il peut être associé. La hantise du cancer doit conduire le clinicien malgache à accélérer autant que possible la confirmation étiologique de tout symptôme respiratoire afin de permettre la prise en charge précoce. De même, la réalisation d'une radiographie thoracique au cours des visites médicales systématiques permettrait le dépistage de nombreux cas précoces encore asymptomatiques. Et le délai de prise en charge pré-hospitalière et hospitalière doit être amélioré.
